# Hederagenin Protects PC12 Cells Against Corticosterone-Induced Injury by the Activation of the PI3K/AKT Pathway

**DOI:** 10.3389/fphar.2021.712876

**Published:** 2021-10-14

**Authors:** Ruohong Lin, Linlin Liu, Marta Silva, Jiankang Fang, Zhiwei Zhou, Haitao Wang, Jiangping Xu, Tiejun Li, Wenhua Zheng

**Affiliations:** ^1^ Center of Reproduction, Development and Aging and Institute of Translation Medicine, Faculty of Health Sciences, University of Macau, Taipa, Macao, China; ^2^ School of Pharmaceutical Sciences, Southern Medical University, Guangzhou, China; ^3^ Research and Development Department, Lansson Bio-Pharm Co., Ltd., Guangzhou, China

**Keywords:** hederagenin, corticosterone, PC12 cells, PI3K, Akt, pathway, depression

## Abstract

Depression is a prevalent psychiatric disorder and a leading cause of disability worldwide. Despite a variety of available treatments currently being used in the clinic, a substantial proportion of patients is unresponsive to these treatments, urging the development of more effective therapeutic approaches. Hederagenin (Hed)*,* a triterpenoid saponin extracted from *Fructus Akebiae,* has several biological activities including anti-apoptosis, anti-hyperlipidemic and anti-inflammatory properties. Over the years, its potential therapeutic effect in depression has also been proposed, but the information is limited and the mechanisms underlying its antidepressant-like effects are unclear. The present study explored the neuroprotective effects and the potential molecular mechanisms of Hederagenin action in corticosterone (CORT)-injured PC12 cells. Obtained results show that Hederagenin protected PC12 cells against CORT-induced damage in a concentration dependent manner. In adittion, Hederagenin prevented the decline of mitochondrial membrane potential, reduced the production of intracellular reactive oxygen species (ROS) and decreased the apoptosis induced by CORT. The protective effect of Hederagenin was reversed by a specific phosphatidylinositol-3-kinase (PI3K) inhibitor LY294002 and AKT (also known as protein kinase B) inhibitor MK2206, suggesting that the effect of Hederagenin is mediated by the PI3K/AKT pathway. In line with this, western blot analysis results showed that Hederagenin stimulated the phosphorylation of AKT and its downstream target Forkhead box class O 3a (FoxO3a) and Glycogen synthase kinase-3-beta (GSK3β) in a concentration dependent manner. Taken together, these results indicate that the neuroprotective effect of Hederagenin is likely to occur via stimulation of the PI3K/AKT pathway.

## Introduction

Depression is a major mental disorder and a leading cause of disability worldwide. The World Health Organization estimates that more than 264 million individuals are affected by depression around the world which poses a substantial socioeconomical burden (James et al., 2018). At present, selective serotonin reuptake inhibitors (SSRIs), as well as serotonin and norepinephrine reuptake inhibitors (SNRIs), are the most commonly used drugs in the clinical treatment of depression (Dale et al., 2015; Gałecki et al., 2018). However, as multiple pathogenic factors are involved in its etiology, a considerable part of patients shows no improvements upon treatment. This is further aggravated by the fact that these antidepressant medications are often associated with a variety of side effects such as sexual dysfunction, hypertension, sleep and gastrointestinal disturbances and higher fracture risk (Predictable et al., 2006; Vestergaard et al., 2006; Cascade et al., 2009). Thus, the search for new effective antidepressants with no or low adverse effects is desirable to improve the therapeutic arsenal. In this regard, the use of alternative natural compounds derived from medicinal plants has been increasingly attracting attention all over the world due to their lower toxicity, adverse events, and better compliance.

Hederagenin (Hed, C_30_H_48_O_4_) is a pentacyclic triterpenoid saponin, widely found in various plants and that is used as a chemotaxonomic marker of the Sapindaceae family. Currently, there is an increasing interest in Hederagenin due to its multiple pharmacological activities including anti-hyperlipidemia, anti-inflammatory (Takagi et al., 1980; Su-Hong et al., 2015), as well as significant cytotoxic effects in several types of cancers (Kim et al., 2017a; Bai et al., 2017; Tatia et al., 2019). In addition, hederagenin is also able to promote the degradation of neurodegenerative mutant disease proteins (Wu et al., 2017) and may act as a therapeutic candidate for Alzheimer’s disease (Wang et al., 2020) and ischemic stroke (Yu et al., 2020). Recently, Hederagenin was reported to have antidepressant-like effects (Zhou et al., 2010; Jin et al., 2012; Liang et al., 2015). However, the mechanisms by which hederagenin exerts its antidepressant-like are not clear, and further proofs regarding the protective activity of Hederagenin against depression models are necessary.

In spite of the pathophysiology of depression not being fully understood, several putative mechanisms have been suggested. One well-known mechanism is associated with the overactivation of the hypothalamic–pituitary–adrenal (HPA) axis, which is characterized by an increase of the concentration of circulating glucocorticoids. The elevated glucocorticoids levels lead to DNA damage in nerve cells and eventually induce apoptosis and depression-like behaviors (Latt et al., 2018).

PC12 cells, a cell line derived from a pheochromocytoma of the rat adrenal medulla, are widely used to examine mechanistic studies of neuronal functions due to their typical neuronal features (Greene and Tischler, 1976). Importantly, numerous classical antidepressants, such as venlafaxine (Wang et al., 2013) and fluoxetine (Zeng et al., 2016) have shown protective effects against the cytotoxicity induced by the glucocorticoid corticosterone (CORT) in PC12 cells. These evidence indicate that CORT-treated PC12 cells is a valid depression cellular model to evaluate the efficacy of antidepressant candidates and to explore the mechanisms of action of these compounds. Accordingly, if neurons can be protected from glucocorticoid-induced injury, the neurological dysfunction caused by chronic stress may be alleviated.

The effects of Hederagenin against CORT-induced damage and the mechanisms underlying these responses remain unclear. Therefore, in the present study, we used this model to study the protective effect of Hederagenin and its potential mechanisms of action. Obtained results demonstrated that Hederagenin inhibited CORT-induced injury through the activation of the AKT/FoxO3a/GSK3β pathway. These findings provide a novel insight into the molecular mechanisms by which Hederagenin protects neurons against CORT-induced damage.

## Materials and Methods

### Materials

Hederagenin (≧98% purity by HPLC) was purchased from Nanjing Puyi Biological Biotech Co. Ltd. (Nanjing, China). CORT (purity ≧98%) was purchased from Meilunbio (Dalian, China). Fetal bovine serum (FBS), Dulbecco’s Modified Eagle Medium, penicillin and streptomycin were purchased from Gibco (Grand Island, United States). Primary antibodies for GAPDH, anti-phospho-AKT, anti-AKT, anti-phospho-FoxO3a, anti-FoxO3a, anti-phosphor-GSK3β and anti-GSK3β were purchased from Cell Signaling (CST, United States), Brain-derived neurotrophic factor (BDNF) was purchased from Abcam (Cambridge, United Kingdom). Dimethyl sulfoxide (DMSO) was purchased from Sigma-Aldrich Co. (St Louis, United States). 3-(4,5-dimethylthiazol-2-yl)-2,5-diphenyl tetrazolium bromide (MTT) was purchased from Molecular Probes (Eugene, OR, USA). LY294002 was purchased from Calbiochem. MK-2206 was purchased from Selleckchem. Annexin V FITC Apoptosis Detection Kit was obtained from BD Biosciences (San Diego, CA) The commercial kit for measuring Reactive Oxygen Species (ROS) and mitochondrial membrane potential ∆Ψm (JC-1) content were purchased from Beyotime Biotechnology (Shanghai, China).

### Methods

#### Cell Culture

PC12 cells were kindly provided by Dr. Gordon Guroff (National Institute of Child Health and Human Development, National Institutes of Health, Bethesda, MD). Cells were cultured in 25-cm^2^ flasks in Dulbeccoʼs modified Eagleʼs medium (DMEM), supplemented with 10% FBS, 100 μg/ml streptomycin, 100 U/ml penicillin, and incubated at 37°C with 5% CO_2_ humidified atmosphere. The cells were sub-cultured 2–3 times a week at a split of 1:5 with fresh medium as described above.

#### Hippocampal Neurons Primary Culture

Primary cultured neurons were prepared from C57BL/6 1 day born mice (weighing 4–6 g) provided by the Animal Facility of the University of Macau, as previously described (Zhao, 2019). Briefly, the mice were disinfected with 75% alcohol, the brain was collected and the whole hippocampus tissue was dissected and washed with HBSS three times to remove the mixed blood vessels. Subsequently, the hippocampus was cut into small pieces, washed with HBSS and digested with 0.125% trypsin in a CO_2_ incubator for 15 min. After stopping the digestion with 10% FBS, the cells were centrifuged at 1,000 rpm for 10 min and suspended in a chemically defined Neurobasal medium supplemented with 2% B27. The cells were seeded in poly-D- lysine treated plates at a density of 2×10^5^ cells/ml, and grown at 37°C with 5% CO_2_ humidified atmosphere. Half of medium was changed every 2 days. Experimental treatments were performed when cell density reached 70% confluency.

#### Cell Treatments

Hederagenin and CORT were dissolved in DMSO and the final concentration of DMSO was taken up to less than 0.1% for all treatments. The same volume of DMSO (0.1% v/v) was added to the control groups. To research the neuroprotective effect of Hederagenin, PC12 cells were divided into the following three groups: 1) Control group: cells were cultured for 48 h in DMEM; 2) CORT model treatment group: cells were incubated for 48 h in DMEM in the presence of 100, 200, 400, 600 or 800 µM CORT; 3) CORT plus Hederagenin treatment group: cells were incubated for 48 h in DMEM containing 400 µM CORT in the presence of 0.03, 0.1, 0.3 or 1 µM Hederagenin.

#### MTT Assay

Cell viability was assessed by MTT assay as previously described with some modifications (Zheng and Quirion, 2009). Briefly, PC12 cells were seeded in 96-well plates at a density of 1×10^4^ cells/well in DMEM medium supplemented with 1% FBS. After cell adhesion to the plates, the cultures were treated with Hederagenin and CORT for 48 h. Subsequently the cells were incubated with MTT (0.5 mg/ml) for 3–4 h. After this period the medium was removed and DMSO (150 μl) was added to each well. Absorbance was detected at 490 nm by Infinite M200 PRO Multimode Microplate (Tecan, Switzerland). The OD values obtained for each group were averaged and cell viability was presented as a percentage of the control group.

#### Annexin V-FITC/Propidium Iodide Staining for Apoptosis Evaluation

CORT-induced apoptosis was determined using the Annexin V-FITC/PI staining method according to the manufacturer’s protocol (BD Biosciences, CA). Briefly, after appropriate treatment, PC12 cells were harvested, washed with ice-cold PBS and resuspended in 195 µl annexin V-FITC binding buffer supplemented with 5 µl Annexin V-FITC solution for 15 min at room temperature in the dark. The buffer was then replaced by 190 µl binding buffer PI (10 μl) and apoptotic cells were immediately analyzed by flow cytometry. According to the assay principles, viable cells are both Annexin V and PI negative (lower-left quadrant), early apoptotic cells are Annexin V positive and PI negative (lower-right quadrant), and late apoptotic or necrotic cells are both Annexin V and PI positive (upper-left quadrant). CellQuest ™ Pro Software (BD Biosciences, San Diego, CA) was used to quantify apoptotic cells and the apoptosis rate was expressed as the percentage of Annexin V-positive cells.

#### Measurement of Intracellular Reactive Oxygen Species Levels

Intracellular ROS levels were determined using a ROS assay kit according to the manufacturer’s protocols (Beyotime, China). Briefly, after appropriate treatment, PC12 cells were incubated with the diluted green-fluorescent probe DCFH-DA (10 µM in medium) for 30 min in the dark. Then the cells were rinsed with serum-free DMEM and the fluorescence was observed and recorded using a fluorescence microscope (EVOS FL Imaging System). All values were normalized to the control group.

#### Measurement of Mitochondrial Membrane Potential

JC-1 detection kit was used to monitor the alterations in mitochondrial membrane potential (Ψm) according to the manufacturer’s protocols. In brief, after appropriate treatment, PC12 cells were washed with serum-free DMEM and stained with JC-1 (10 μg/ml in medium) at 37°C for 20 min in the dark. Subsequently, cells were washed twice with ice-cold JC-1 staining buffer and the fluorescent signal in the cells was directly observed and photographed using a fluorescence microscope (EVOS FL Imaging System). The ratio (%) of red/green fluorescence intensity was calculated by ImageJ software as a percentage of the control group.

#### Western Blot Analysis

Western blot was performed as previously described (Zheng and Quirion, 2009). Briefly, harvested cells were washed with ice-cold PBS and lysed in RIPA buffer. Protein concentration was quantified with a BCA protein assay kit according to the manufacturer’s instructions. Equal amounts of total protein (20 µg) were separated by 8–12% SDS-PAGE and transferred to PVDF membranes. The membranes were blocked with 5% BSA in TBST for 1 h at room temperature and then incubated with diluted selected primary antibodies at 4°C overnight. The membranes were then washed three times with TBST and further probed with the corresponding HRP-conjugated secondary antibodies for 1 h at room temperature. The unbound secondary antibodies were finally washed with TBST. Bands were visualized using enhanced chemiluminescence (ECL). The intensity of the bands was semi-quantified using Image J software (National Institute of Health).

#### Statistical Analysis

Statistical analysis was performed using GraphPad Prism 5.0 statistical software (GraphPad software, Inc.). The experiments were performed in triplicates. The data is expressed as mean ± standard deviation (SD). Statistical analysis was carried out using one-way ANOVA followed by Tukey’s multiple comparison post-hoc test. Differences were considered to be statistically significant at *p* < 0.05.

## Results

### Hederagenin Alleviated CORT-Induced Injury in PC12 Cells

**FIGURE 1:**
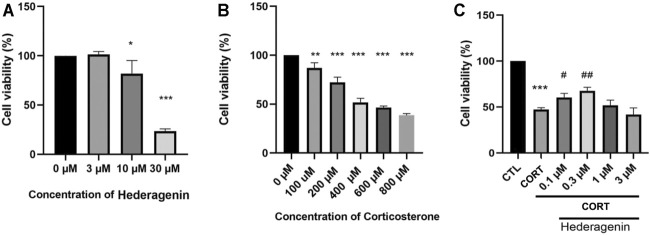
Hederagenin attenuated the decrease in cell viability induced by corticosterone (CORT) in PC12 cells. **(A)** The cytotoxicity of Hederagenin. PC12 cells were treated with Hederagenin (3–30 μM) for 48 h, then cell viability was measured using the MTT assay. **(B)** The cytotoxicity of CORT. PC12 cells were treated with CORT (100–800 μM) for 48 h, and cell viability was measured by MTT. **(C)** The protective effect of Hederagenin against CORT-induced damage. PC12 cells were treated with CORT (400 μM) and Hederagenin at the indicated concentrations for 48 h, then cell viability was measured by MTT. The data are represented as the mean ± SD of three independent experiments. *p < 0.05, **p < 0.01, ***p < 0.001 compared with control. #< 0.05, ##< 0.01 compared with CORT group, SD: standard deviation.

Assessment of the neurotoxicity of Hederagenin on PC12 cells revealed that cell viability was not significantly affected by Hederagenin concentrations below 3 μM, although 10 µM Hederagenin significantly reduced cell viability (Figure 1A). Accordingly, Hederagenin range of concentrations lower than 3 µM were used in the subsequent studies. To assess the effect of CORT on cell viability, PC12 cells were treated with increasing concentrations of CORT (100, 200, 400, 600 and 800 µM) or vehicle (0.1% DMSO). Figure 1B shows that CORT decreased cell viability in a concentration-dependent manner, with 400 µM causing a decrease of approximately 50% of viable cells in comparison with the control group. Therefore, the concentration 400 µM was used in the subsequent experiments to induce the CORT-induced cell injury model (Figure 1B). To determine the cytoprotective effects of Hederagenin against CORT-induced injury, PC12 cells were treated with 400 μM of CORT in the absence or presence of different doses of Hederagenin for 48 h. Obtained results showed that incubation with Hederagenin (0.1 and 0.3 µM) significantly increased the survival rate to 65 and 72%, respectively (Figure 1C). These results indicate that Hederagenin is able to protect PC12 cells against CORT-induced cellular injury.

### Hederagenin Attenuated the Loss of Mitochondrial Membrane Potential in CORT-Treated PC12 Cells

**FIGURE 2:**
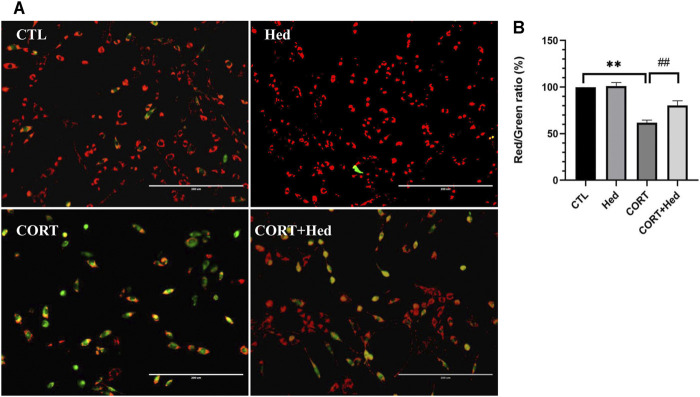
Hederagenin attenuated CORT-induced mitochondrial membrane potential (ψm) loss in PC12 Cells. **(A)** PC12 cells were treated with Hederagenin (0.3 μM) and CORT (400 μM) for 48 h, then Δψm was determined by the JC-1 assay. The decline in the membrane potential was reflected by the shift of fluorescence from red to green indicated by JC-1. **(B)** Quantitative analysis of **(A)**. CTL, non-treated control; CORT, (400 µM) CORT treatment; CORT + Hed, (400 μM) CORT plus (0.3 μM) hederagenin treatment; Hed, (0.3 μM) Hederagenin treatment only. Bars represent mean ± SD of three independent experiments. **p < 0.01 compared with CTL group; ##*p* < 0.01 compared with CORT group.

The loss of mitochondrial membrane potential (Δψm) is an early sign of apoptosis and the decline of red to green fluorescence ratio in JC-1 assay is used as an indicative of Δψm loss (Smiley et al., 1991). Assessment of the effect of Hederagenin and CORT on the mitochondrial membrane potential of PC12 cells, revealed that CORT incubation promoted a significant decrease in the red/green fluorescence ratio compared with the control group (*p* < 0.01), which is indicative of a decrease of ΔΨm (Figure 2). In contrast, treatment of cells with Hederagenin at 0.3 μM markedly attenuated the CORT-induced decline of Δψm. Furthermore, 0.3 μM Hederagenin alone had no effect on the ratio of red/green fluorescence (Figure 2). These results indicate that the anti-apoptotic effect of Hederagenin is probably due to its effect on the recovery of the mitochondrial function.

### Hederagenin Blocked CORT-Induced Increase of Intracellular ROS in PC12 Cells

**FIGURE 3:**
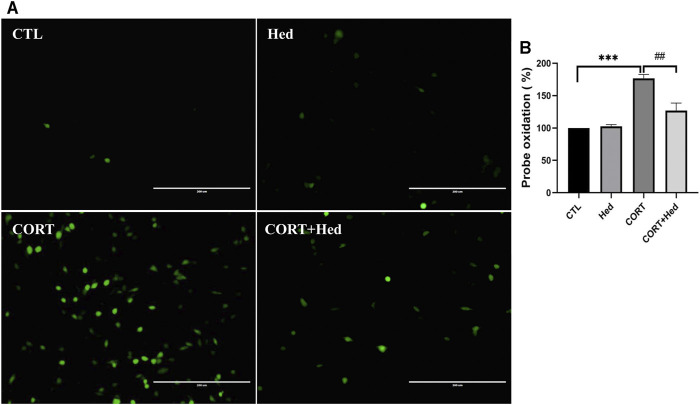
Hederagenin inhibited CORT-induced increase of reactive oxygen species (ROS) levels in PC12 cells. **(A)** PC12 cells were co-treated with Hederagenin (0.3 μM) and CORT (400 μM) for 48 h, then, intracellular ROS levels were measured. **(B)** Quantitative analysis of **(A)**. The data are represented as the mean ± SD of three independent experiments. ***p < 0.001 versus the control group, ##*p* < 0.01 versus the CORT group.

Oxidative stress is a redox state caused by an imbalance between the generation and detoxification of ROS, that contributes to diverse neurological disorders (Simonian and Coyle, 1996). Previous studies indicated that the cytotoxicity of CORT is mediated by increasing levels of ROS (Mao et al., 2011; Jin et al., 2019). Thus, the protective role of Hederagenin against CORT-induced oxidative stress was also investigated. As shown in Figure 3, CORT increased the production of ROS, whereas co-treatment with Hederagenin prevented this increase. Treatment with Hederagenin alone did not affect the production of ROS (Figure 3). These findings suggest that Hederagenin may reduce CORT-induced oxidative stress in PC12 cells by reducing the accumulation of ROS.

### Hederagenin Attenuated CORT-Induced Apoptosis in PC12 Cells

**FIGURE 4:**
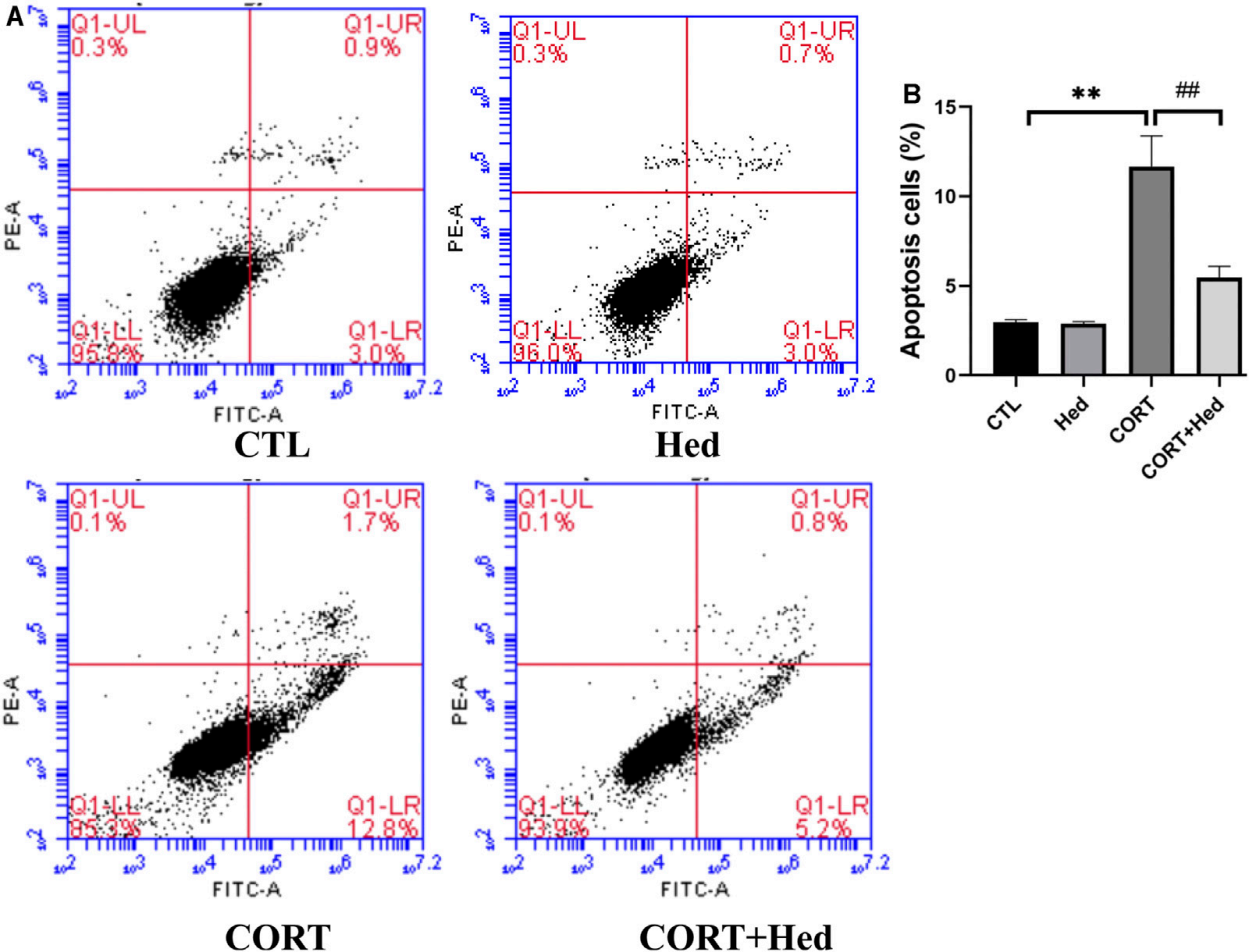
Hederagenin decreased the apoptosis induced by CORT. PC12 cells were co-treated with Hederagenin (0.3 μM) and CORT (400 μM) for 48 h, and cell apoptosis was determined by flow cytometry. **(A)** Representative dot plots of intact cells at lower-left quadrant, FITC(-)/PI(-); early apoptotic cells at lower-right quadrant, FITC(+)/PI(-) and late apoptotic or necrotic cells at upper-right quadrant, FITC(+)/PI(+). **(B)** Quantitative analysis of **(A)**. The data are represented as the mean ± SD of three independent experiments. **p < 0.01 versus the control group, #*p* < 0.05 versus the CORT group.

Annexin V-FITC and PI double staining was performed to investigate the protective effect of Hederagenin against CORT-induced cell apoptosis. Obtained results, show that CORT exposure significantly increased early apoptosis, that was prevented by co-treatment with Hederagenin (0.3 μΜ) (Figure 4). Hederagenin treatment alone did not change the apoptosis rate of PC12 cells. The results indicate that Hederagenin is able to inhibit CORT-induced apoptosis.

### Hederagenin Stimulated PI3K/AKT/FoxO3a Signaling

**FIGURE 5:**
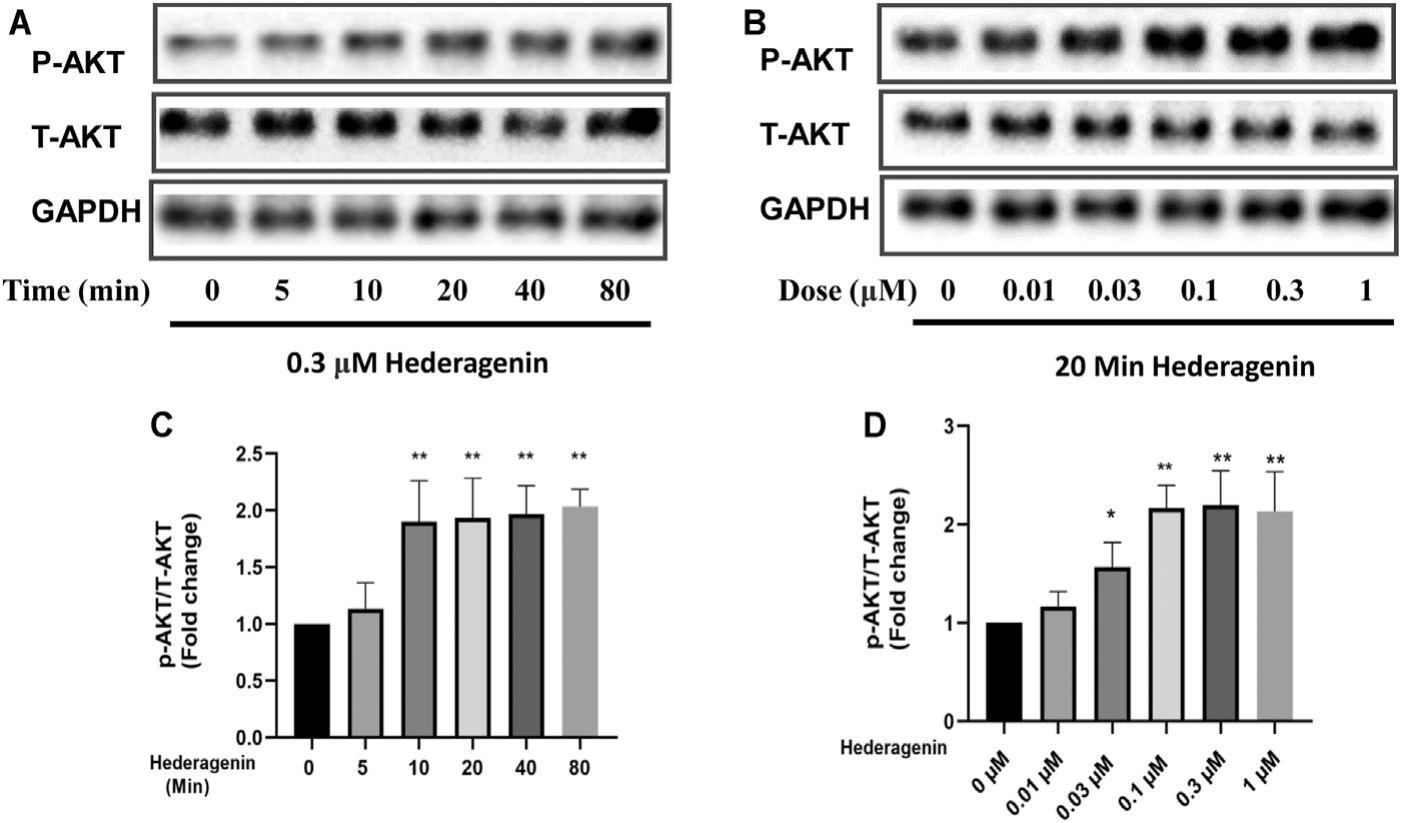
Hederagenin stimulated the phosphorylation of AKT in PC12 cells. **(A,B)** PC12 cells were collected after Hederagenin treatment for different times (0, 5, 10, 20, 40 and 80 min) at 0.3 μM, and at different concentrations (0, 0.01, 0.03, 0.1, 0.3, and 1 μM) for 20 min. The expression of phosphorylated AKT, total AKT and GAPDH were detected by Western blot analysis. **(C,D)** Quantitative analysis of **(A,B)**. The data are represented as the mean ± SD of three independent experiments. *p < 0.05, **p < 0.01, compared with control.

**FIGURE 6:**
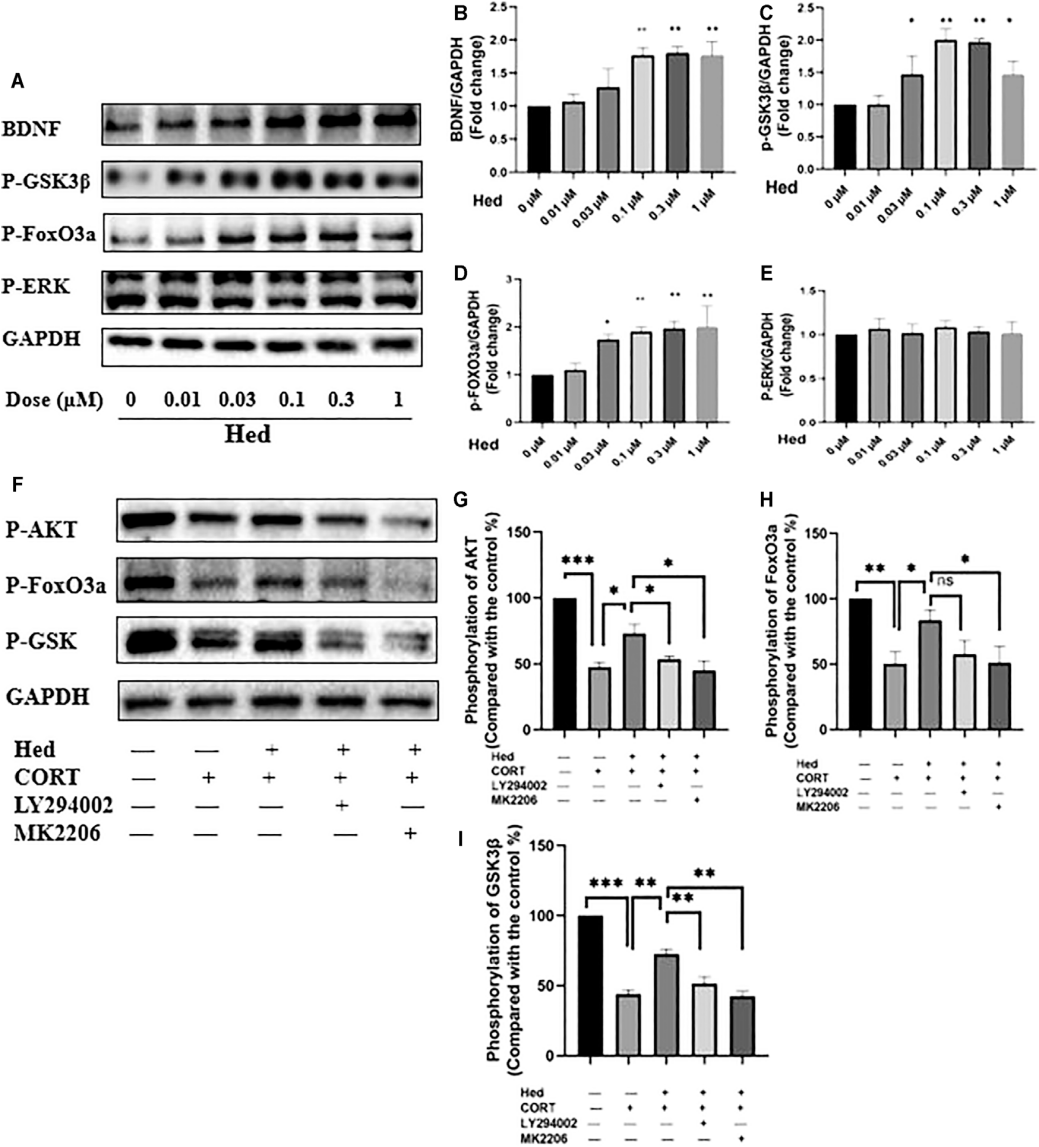
Hederagenin stimulated the phosphorylation of FoxO3a/GSK3β and augmented the expression of BDNF in PC12 cells. **(A)** PC12 cells were collected after Hederagenin treatment at different concentrations, then western blot assays were performed to detect the levels of BDNF and the phosphorylation of FoxO3a, GSK-3β and ERK. **(B–E)** Quantitative analysis of **(A)**. **(F)** PC12 cells were pre-treated with 20 µM LY294002 and 5 µM MK2206 for 45 min and then incubated with Hederagenin and CORT for 24 h, then the expression of phosphorylated AKT, FoxO3a, GSK-3β and GAPDH was detected by western blot. **(G–I)** Quantitative analysis of **(F)**. The data are represented as the mean ± SD of three independent experiments. *p < 0.05, **p < 0.01 and ***p < 0.001 compared with control group.

Next, we investigated the signaling pathways involved in the protective effect of Hederagenin. Accumulating evidence indicate that AKT and extracellular signal-regulated kinase (ERK) pathways are the major pathways associated with cell survival (Beaulieu, 2012; Kitagishi et al., 2012). Therefore, we explored the effect of Hederagenin on the activation of these signaling pathways on PC12 cells. As shown in Figures 5A–D, Hederagenin stimulated the phosphorylation of AKT in a time- (0.3 µM) and concentration-dependent (20 min) manner. Furthermore, it also promoted an increase in the phosphorylation of AKT two major downstream targets FoxO3a and GSK3β, as well as an upregulation of brain-derived neurotrophic factor (BDNF), in a concentration dependent manner, while having no significant effect on ERK phosphorylation (Figures 6A–E). Western blot assessment of the effect of a PI3K inhibitor LY294002 and an AKT specific inhibitor MK2206 on the phosphorylation cascades, showed that blocking the AKT signaling pathway reduced AKT activation in cells treated with Hederagenin and CORT (Figures 6F–I).

### AKT Signaling Was Involved in the Protective Effect of Hederagenin

**FIGURE 7:**
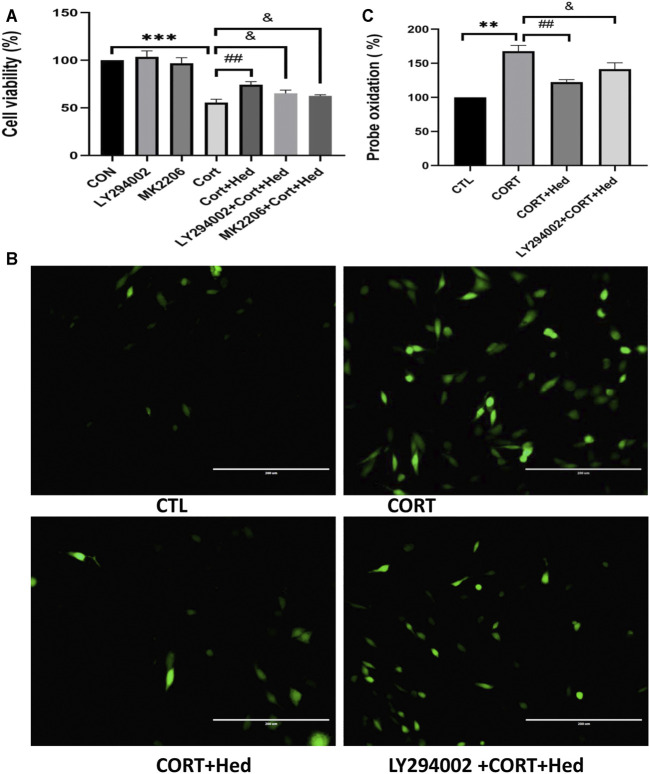
AKT signaling is involved in the protective effects of Hederagenin on PC12 cells against CORT-induced oxidative damage. **(A)** PC12 cells were pretreated with 20 μM LY294002 for 45 min followed by treatment with Hederagenin with or without 400 μM CORT for another 48 h. Then, cell viability was measured by MTT assay. Data represent mean ± SD, ***p < 0.001 versus control group, ##*p* < 0.01 and ^&^ *p* < 0.05 versus CORT-treated group. **(B)** PC12 cells were pretreated with 20 μM LY294002 for 45 min followed by treatment with Hederagenin with or without 400 μM CORT for another 48 h. Then, ROS levels were measured. **(C)** Quantitative data of **(B)**, Data represent mean ± SD of three independent experiments, **p < 0.01 versus control group, ##*p* < 0.01, ^&^ *p* < 0.05 versus CORT group.

Further confirmation of the involvement of PI3K/AKT signaling in the protective effect of Hederagenin, showed that both LY294002 and MK2206 blocked the protective effect of Hederagenin against CORT-induced cell death (Figure 7A). Similar results were obtained from ROS assay, in which Hederagenin failed to suppress the increase of intracellular ROS levels caused by CORT in the presence of LY294002 (Figures 7B,C).

### Neuroprotective Effects of Hederagenin Against CORT-Induced Injury in Primary Hippocampal Neurons

**FIGURE 8:**
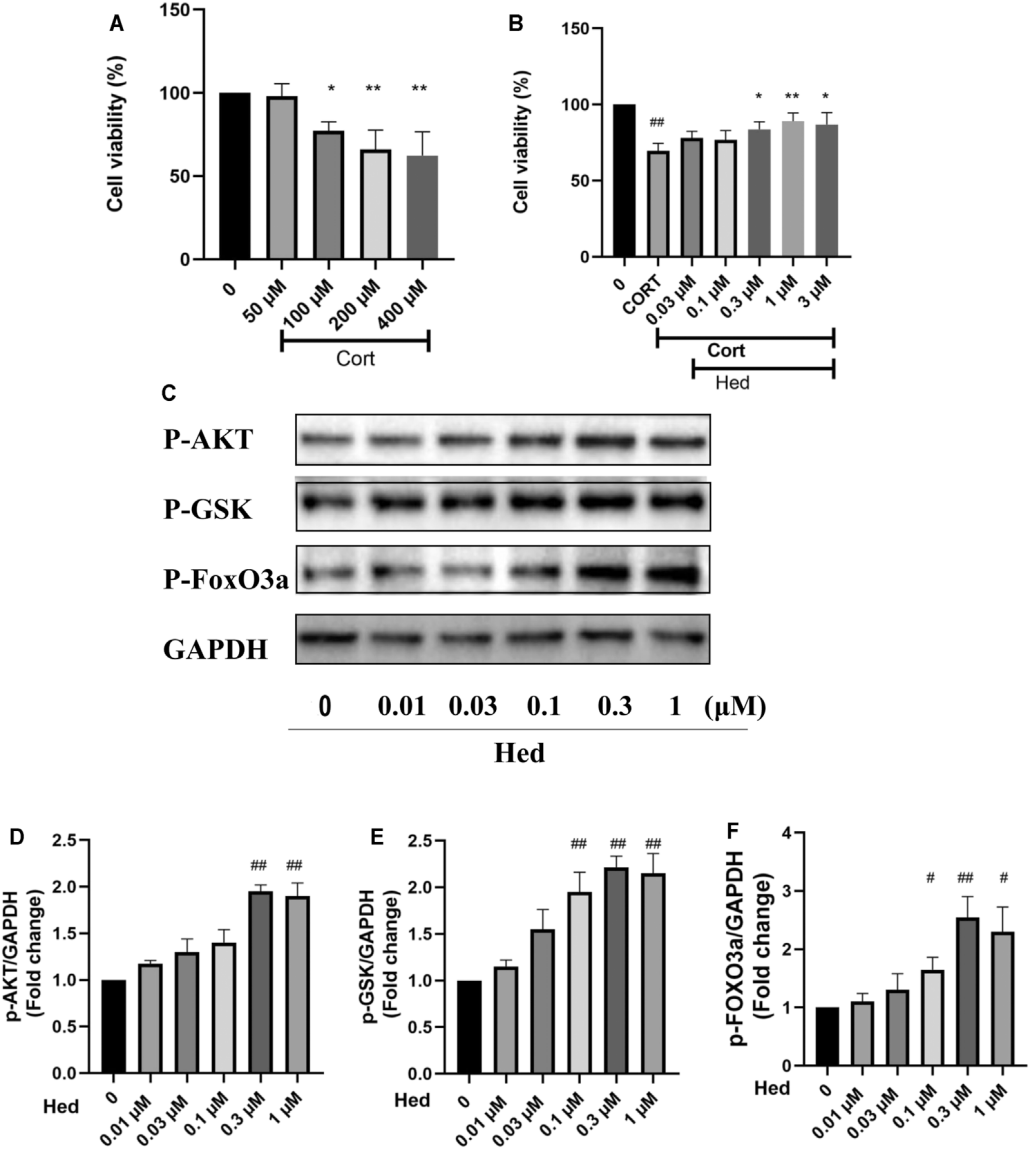
Hederagenin protected primary cultured hippocampal neurons against CORT-induced damage. **(A)** Cytotoxicity of CORT on primary hippocampal neurons. **(B)** Primary hippocampal neurons were treated with CORT (200 μM) and Hederagenin at the indicated concentrations for 48 h, then cell viability was measured by MTT. **(C)** Primary cultured hippocampal neurons were collected after Hederagenin treatment at the indicated concentrations (0, 0.01, 0.03, 0.1, 0.3, and 1 μM) for 20 min and then the expression of phosphorylated AKT, GSK, FoxO3a and GAPDH was detected by western blot. **(D–F)** Quantitative analysis of **(C)**. The data are represented as the mean ± SD of three independent experiments. ^##^ *p* < 0.01 versus CTL group, *p < 0.05 and **p < 0.01 versus CORT group.

To check if the neuroprotective effect of Hederagenin against CORT-induced toxicity is not only applicable to PC12 cells line, the neuroprotective effect of hederagenin on primary hippocampal neurons was also examined. As shown in Figure 8, Hederagenin successfully protected hippocampal neurons from the deleterious effects of CORT in a concentration-dependent manner. Its protection was significant at 0.3 µM and reached its maximum at about 1 μM. Western blot results also showed that Hederagenin increased the phosphorylation of AKT, GSK, FoxO3a in a concentration dependent manner (Figures 8C–F).

## Discussion

**FIGURE 9:**
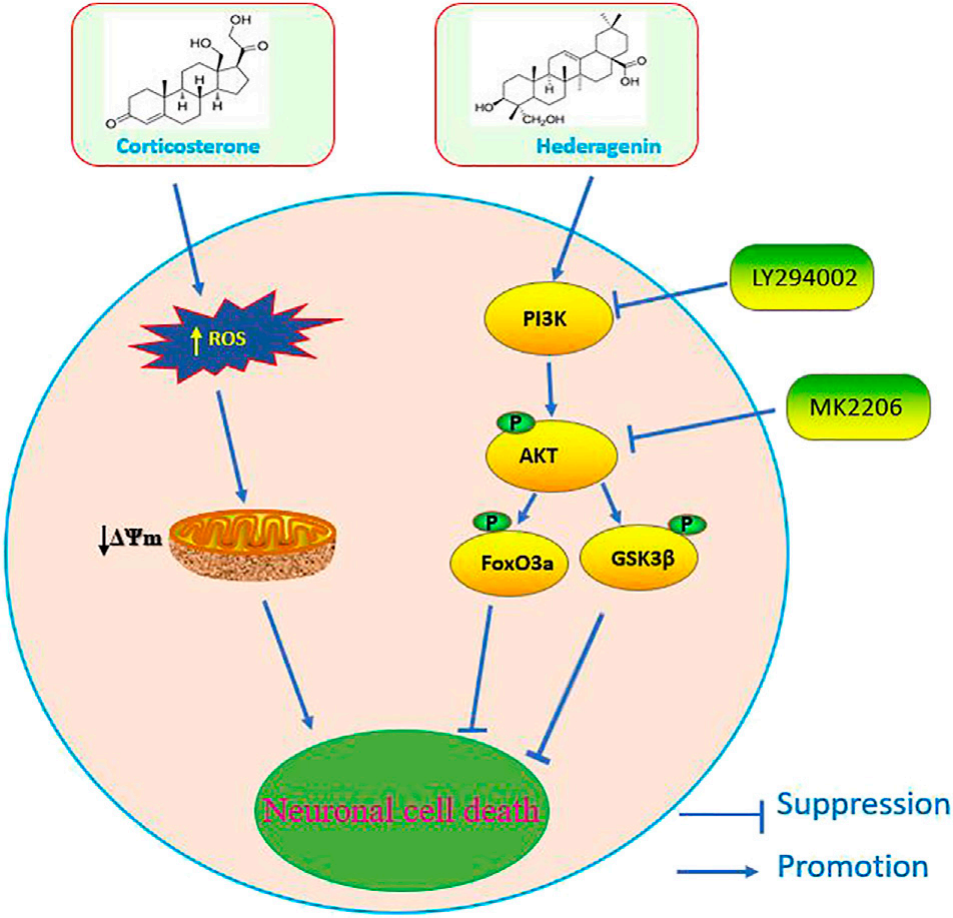
Schematic diagram of the signaling mechanisms involved in the effect of Hederagenin against corticosterone-induced damage.

Depression is a leading cause of disability worldwide, and is becoming a major source of global disease burden (WHO, 2008). Despite the available pharmacological treatments, most antidepressants are only effective in the treatment of some patients and are often associated with a variety of side effects. Therefore, it is urgent to develop more effective therapeutic approaches with no or low adverse effects. Hederagenin, a pentacyclic triterpenoid found in many plants, reported to be able to cross the blood-brain barrier (Yang et al., 2011) was also shown to have some anti-depression-like properties (Jin et al., 2012; Liang et al., 2015). However, the information related to it and the underlying mechanisms is very limited. The present study shows that this natural compound has a significant anti-depression-like action in a classical cellular model of depression that consists in the incubation of PC12 cells and primary cultured neurons with CORT. We report that Hederagenin protected neuronal cells from CORT-induced injury in a concentration dependent manner. In addition, it promoted an upregulation of BDNF levels and the phosphorylation of AKT and its downstream signaling targets FoxO3a/GSK3β, suggesting that Hederagenin action is likely to be mediated via Akt/FoxO3a/GSK3β pathway. A schematic representation of our findings is shown in Figure 9. To our knowledge, this is the first report showing that Hederagenin prevented CORT-induced neuronal injury via this signaling pathway.

In this study, incubation of PC12 cells with 400 μM CORT resulted in a significant decline of the cell survival rate in comparison with untreated control group. Increasing evidence suggest that the cellular mechanisms of CORT-induced insults in PC12 cells involves the induction of mitochondrial dysfunction (Li et al., 2014; Wen-Xia et al., 2019), the overproduction of ROS (Liu et al., 2014b; Zhou et al., 2017) and the induction of apoptosis (Li et al., 2003b; Crochemore et al., 2005). Elucidation of the potential protective action of Hederagenin against these insults showed that it is able to restore the alterations in mitochondrial membrane potential and to downregulate ROS levels increase induced by CORT. These findings suggest that hederagenin anti-apoptotic and cytoprotective effect may occur via the blockade of mitochondrial dysfunction and the decrease of ROS levels.

PI3K/AKT pathway plays a critical role in neuronal cell proliferation and survival (Brunet et al., 2001). AKT is phosphorylated by PDK1/2 and the phosphorylation of AKT activates this kinase. Activated AKT then phosphorylates its downstream proteins, such as GSK3β and FoxO3a transcription factors, controlling cell growth, differentiation and cell survival (Pap and Cooper, 1998; Zhang et al., 2011). FoxO3a belongs to a large family of fork head transcription factors and is largely controlled by posttranslational modifications such as phosphorylation. In the presence of survival factors, PI3K signaling cascade is activated and activated AKT phosphorylates FoxO3a (Brunet et al., 1999), resulting in its relocation from the nucleus to the cytosol inhibiting the induction of the expression of death genes, leading to the survival of cells (Biggs et al., 1999). Previous studies reported that antidepressants treatment promoted an upregulation of the phosphorylation levels of FoxO3a and AKT in PC12 cells (Wang et al., 2013; Zeng et al., 2016; Zeng et al., 2017). Similarly, in this study, Hederagenin treatment also remarkably increased the phosphorylation of AKT and FoxO3a. Furthermore, our MTT results indicate that Hederagenin protective effect was abolished when the cells were treated with the PI3K inhibitor LY294002 or AKT inhibitor MK2206. Similar results were observed in the ROS assay, in which LY29402 inhibited Hederagenin antioxidant action and prevented the reduction of CORT-induced ROS overproduction. These results suggest that Hederagenin protective effect is likely to be mediated via the PI3K/Akt pathway. Nevertheless, since both LY29402 and MK2206 did not show a complete inhibition of the effect of Hederagenin, the possibility of more signaling pathways, other than the PI3K/AKT, being involved in the beneficial effect of Hederagenin cannot be ruled out. Previous studies have shown that Hederagenin is not only able to promote the activation of AKT pathway but also the ERK pathway in hepatocytes (Kim et al., 2017b). However, in the present study, phospho-ERK signaling was not significantly affected by Hederagenin treatment.

BDNF, a key neurotrophin in the brain, is a pivotal regulator of neuronal survival, hippocampal neurogenesis, and behavioral effects of antidepressants (Lee et al., 2002; Shirayama et al., 2002; Scharfman et al., 2005). It has been widely reported that depression is associated with a downregulation of brain BDNF levels (Brunoni et al., 2008; Lee and Kim, 2010) being regarded as a major target of antidepressant medications (Chen et al., 2001). An accumulating amount of evidence indicate that the neuro-modulatory effects of BDNF can be induced by various classes of antidepressants, such as selective serotonin reuptake inhibitors, norepinephrine selective reuptake inhibitors, monoamine oxidase inhibitors (Duman and Monteggia, 2006); and medicinal plant compounds such as resveratrol, curcumin and lignan glycoside (Huang et al., 2011; Liu et al., 2014a; Yang et al., 2020). Consistent with these findings, our results revealed that BDNF expression was significantly increased after treatment with Hederagenin, suggesting that BDNF is involved in its antidepressant-like effect. Besides, it has been reported that BDNF can stimulate the phosphorylation of FoxO3a by activation of the PI3K/AKT pathway in human neuroblastoma SH-SY5Y cells (Mai et al., 2002; Zhu et al., 2004) and a similar regulatory effect was found on primary cultured neurons in our previous studies (Zheng et al., 2002; Zheng and Quirion, 2004). Taken together, these findings suggest that Hederagenin can activate the FoxO3a/PI3K/AKT pathway by increasing the levels of BDNF.

GSK3β, an important intracellular protein kinase, is among the most extensively studied key downstream substrates of PI3K/AKT survival signaling pathway. GSK3β activity is restrained upon Ser9 phosphorylation by AKT (Cross et al., 1995) and its inhibition is one of the key mechanisms by which PI3K/AKT stimulates neuronal survival (Hetman et al., 2000). Besides evidence of GSK3β important regulatory effects in neuronal survival, increasing reports suggest its involvement in the pathophysiological mechanisms of depression, as GSK3β inhibitors have anti-depressant effects and ameliorate depressive-like behavior in animal models (Kaidanovich-Beilin et al., 2004). In addition, treatment with antidepressants was shown to promote the upregulation of phospho-Ser9-GSK3β expression levels in animal models (Li et al., 2004a; Beurel et al., 2011; Garza et al., 2012). In the present study, the increase of AKT phosphorylation after Hederagenin treatment is consistent with the enhancement of GSK3β phosphorylation. Interestingly, BDNF can also increase the phosphorylation of GSK3β, which therefore inhibits GSK3β activity (Mai et al., 2002). Thus, the possible signal-transduction mechanisms underlying Hederagenin regulation of GSK3β activity may involve the upregulation of BDNF levels and the activation of AKT signaling, which eventually contribute to increased neurogenesis and survival.

Since depression has been associated with the over-activation of the hypothalamic-pituitary-adrenal axis that promotes the increase of corticosterone serum levels. The cell model induced by CORT is a canonical *in vitro* model for screening compounds with potential anti-depressive-like activities (Li et al., 2003a; Li et al., 2004b; Mao et al., 2012; Wang et al., 2013; Zeng et al., 2016; Tian et al., 2018). We find that hederagenin is a promising candidate for the treatment of depression in present study using PC12 cells treated with CORT. This finding is consistent with the animal study showing that HG significantly increased the percent of sucrose preference in the sucrose preference test and decreased the immobility time in the forced swimming test (Liang et al., 2015). More important is our present finding about the involvement of PI3K/Akt/FoxO3a, GSK3β, and BDNF, is new and have not been investigated including in animal models of depression. These data will add immense value to the pre-clinical evaluation of hederagenin. Moreover, as depression is a complicated mental disorder, it is beneficial to evaluate the anti-depressant effects of hederagenin in different cellular and animal models. Further investigations are also needed to compare the effects of hederagenin on the model of CORT-induced PC12 cell toxicity with other *in vitro* and *in vivo* models.

## Conclusion

In conclusion, the present study demonstrates that Hederagenin exerted antidepressant-like and anti-apoptotic effects in an *in vitro* model of depression. We found that the potential mechanism of these effects is at least in part due to hederagenin activation of the PI3K/AKT pathway and through the modulation of mitochondrial disfunction. These results provide valuable evidence on hederagenin neuroprotective effect against CORT-induced damage in the pathogenesis of depression *in vitro*, and serves as a reference for further studies aiming to assess the potential of this natural antidepressant compound in the future. Further clarification of the mechanisms of hederagenin action, may help to elucidate potential applications for this potent neuroprotective candidate.

## Data Availability

The original contributions presented in the study are included in the article/Supplementary Material, further inquiries can be directed to the corresponding author.
